# Mortality within three months after nonfatal ischemic stroke treated by mechanical thrombectomy in routine care—data from the German Stroke Registry

**DOI:** 10.1186/s42466-025-00427-7

**Published:** 2025-10-01

**Authors:** Marianne Hahn, Sonja Gröschel, Livia Sophie Lang, Ahmed E. Othman, Klaus Gröschel, Timo Uphaus

**Affiliations:** 1 Department of Neurology, University Medical Center of the Johannes Gutenberg University Mainz, Langenbeckstr. 1, 55131 Mainz, Germany; 2 Department of Neuroradiology, University Medical Center of the Johannes Gutenberg University Mainz, Langenbeckstr. 1, 55131 Mainz, Germany; 3 Department of Neurology, University Hospital, RWTH Aachen University, Aachen, Germany; 4 Department of Neuroradiology, University Hospital, RWTH Aachen University, Aachen, Germany; 5 Department of Neurology, Charité – Universitätsmedizin Berlin, Berlin, Germany; 6 Department of Neuroradiology, Charité – Universitätsmedizin Berlin, Berlin, Germany; 7 Department of Vascular Neurology, University Hospital Bonn, Bonn, Germany; 8 Department of Neuroradiology, University Hospital Bonn, Bonn, Germany; 9 Department of Neurology, University Hospital Frankfurt, Frankfurt am Main, Germany; 10 Department of Neuroradiology, University Hospital Frankfurt, Frankfurt am Main, Germany; 11 Department of Neurology, University Medical Center Göttingen, Göttingen, Germany; 12 Department of Neuroradiology, University Medical Center Göttingen, Göttingen, Germany; 13 Department of Neurology, Bezirkskrankenhaus Günzburg, Günzburg, Germany; 14 Department of Neuroradiology, Bezirkskrankenhaus Günzburg, Günzburg, Germany; 15 Department of Neurology, Asklepios Klinik Hamburg Altona, Hamburg, Germany; 16 Department of Neuroradiology, Asklepios Klinik Hamburg Altona, Hamburg, Germany; 17 Department of Neurology, University Medical Center Hamburg-Eppendorf, Hamburg, Germany; 18 Department of Neuroradiology, University Medical Center Hamburg-Eppendorf, Hamburg, Germany; 19 Department of Neurology, Klinikum Hanau, Hanau, Germany; 20 Department of Neurology, Klinikum Nordstadt, Hannover, Germany; 21 Department of Neuroradiology, Klinikum Nordstadt, Hannover, Germany; 22 Department of Neurology, Mühlenkreiskliniken, Johannes Wesling Klinikum Minden, Minden, Germany; 23 Department of Radiology, Mühlenkreiskliniken, Johannes Wesling Klinikum Minden, Minden, Germany; 24 Department of Neurology, Ludwig Maximilian University (LMU), Munich, Munich, Germany; 25 Department of Neuroradiology, Ludwig Maximilian University (LMU), Munich, Munich, Germany; 26 Institute for Stroke and Dementia Research, Ludwig Maximilian University (LMU), Munich, Munich, Germany; 27 Department of Neurology, Klinikum Rechts der Isar, School of Medicine, Technical University of Munich, Munich, Germany; 28 Department of Neuroradiology, Klinikum Rechts der Isar, School of Medicine, Technical University of Munich, Munich, Germany; 29 Department of Neurology, Klinikum Osnabrück, Osnabrück, Germany; 30 Department of Neurology & Stroke, University Hospital Tübingen; Hertie Institute for Clinical Brain Research, University of Tübingen, Tübingen, Germany; 31 Department of Neuroradiology, University Hospital Tübingen, Tübingen, Germany

**Keywords:** Ischemic stroke, Mechanical thrombectomy, Endovascular stroke therapy, Mortality, Complications, Registry studies

## Abstract

**Background:**

Mechanical thrombectomy (MT) is a highly effective treatment for large vessel occlusion (LVO) ischemic stroke. However, a substantial share of patients have lethal outcome within 3 months. Individualization of outcome prognostication is needed to support clinical decision-making throughout the care pathway after MT. We investigate predictors of lethal outcome in patients with nonfatal LVO, defined by discharge alive from primary treating hospital, in a large prospective registry study of MT under routine care conditions.

**Methods:**

6,518 patients with nonfatal LVO treated by MT enrolled in the German Stroke Registry-Endovascular Treatment from May 2015-December 2021 were analysed with regard to lethal outcome by 3 month follow-up. Univariate group comparisons and multiple logistic regression analysis were performed to identify patients with high odds for survival or lethal outcome.

**Results:**

We report 11.6% (757/6,518) 3 month mortality following hospital discharge after LVO treated by MT. Besides better functional outcome at discharge (modified Rankin scale < 4, odds ratio, OR [95% confidence interval, CI]: 2.38 [1.71–3.32], *p* < 0.001; National Institute of Health Stroke scale < 8, OR [95%CI]: 3.45 [2.55–4.66], *p* < 0.001), intravenous thrombolysis (OR [95%CI]: 1.48 [1.17–1.88], *p* = 0.001), successful recanalization (OR [95%CI]: 1.43 [1.08–1.90], *p* = 0.014) and discharge to a neurorehabilitative facility (versus nursing home: OR [95%CI]: 0.39 [0.26–0.58], *p* < 0.001; versus home: OR [95%CI]: 0.69 [0.49–0.97], *p* = 0.032) were independent predictors of survival. Predictors of lethal outcome were older age (OR [95%CI]: 1.09 [1.07–1.10], *p* < 0.001), male sex (OR [95%CI]: 1.24 [1.00–1.55], *p* = 0.049), premorbid disability (OR [95%CI]: 1.47 [1.08–2.02], *p* = 0.016), active smoking (OR [95%CI]: 1.51 [1.06–2.14], *p* = 0.023), anticoagulation therapy prior to LVO (OR [95%CI]: 1.45 [1.09–1.92], *p* = 0.010), stroke etiology, general anaesthesia during MT (OR [95%CI]: 1.31 [1.02–1.69], *p* = 0.035) and intracerebral haemorrhage (OR [95%CI]: 1.50 [1.13–1.99], *p* = 0.005).

**Conclusions:**

Lethal outcome after hospital discharge within 3 months after MT is frequent, accounting for more than one quarter of overall 3-month mortality after MT of LVO. Predictors of survival enable individual outcome prognostication, which assists clinical decision-making with regard to surveillance concerning complications, rehabilitative resource allocation and counselling about goals of care.

**Trial registration:**

ClinicalTrials.gov (Identifier: NCT03356392, Date of registration: 2017/11/22).

**Supplementary Information:**

The online version contains supplementary material available at 10.1186/s42466-025-00427-7.

## Background

Mechanical thrombectomy (MT) is a highly effective treatment for large vessel occlusion (LVO) ischemic stroke that has become standard of care in acute stroke treatment. Despite significant beneficial effects of MT on stroke-associated disability, a relevant share of patients in randomized controlled trials (RCTs) of MT have lethal outcome three months after MT. Mortality rates within RCTs range from 15 to 30% of patients, depending on vessel territory, infarct size and time window of treatment [9, 16, 24, 25]. Under routine care conditions, patients treated with MT significantly differ from trial populations and 3 month mortality is even higher [20, 27, 31]. As a consequence, patients with LVO stroke represent 90% of all acute ischemic stroke deaths [18]. Predictors of mortality after MT of LVO enable individualization of outcome prognostication and support patients, relatives and physicians with regards to clinical-decision making. While in-hospital mortality and cumulative 90-day mortality after MT are well described [6, 7, 14, 17, 19, 28, 29], predictors of 3 month mortality in patients who survive the acute hospital stay are hardly available. However, predictors of mortality in patients with such nonfatal LVO are of great relevance not only to identify patients at high risk for potentially treatable complications after discharge, but also for rehabilitative resource allocation, evaluation of care needs and counselling of patients and relatives regarding goals of care. Outcome prognostication after the acute phase may, in the future, be of even greater relevance than before, since MT may increasingly be performed also in populations with high odds of severe persisting deficits, such as large infarct sizes [13, 24, 25, 30]. Therefore, predictors of lethal outcome after hospital discharge will be important for targeted allocation of resources to meet palliative care needs in these patients [2].

With our study, we aim to investigate 3-month mortality in patients with nonfatal LVO treated by MT (defined by discharge alive from treating hospital) in a nationwide, multicentre, prospective registry study of MT in clinical practice in Germany. Firstly, we aim to quantify and describe patients with lethal outcome after discharge from treating hospital. Furthermore, we aim to identify patient, stroke and treatment characteristics, which may serve as independent predictors for 3-month mortality after nonfatal MT-treated LVO in order to assist individualization of outcome prognostication in clinical practice.

## Methods

### Study population

The German Stroke Registry—Endovascular Treatment (GSR-ET) is an ongoing academic, independent, prospective, multicentre, observational registry study. Thirty certified German stroke centres consecutively enroll adult patients diagnosed with acute ischemic stroke due to LVO and intention to be treated with MT. Baseline demographics, comorbidities, clinical and procedural information as well as clinical follow-up after 90 days are recorded. More detailed information on the registry’s study protocol and variables has been published before [1]. The study cohort consisted of all patients discharged alive after MT of LVO. Therefore, of the 13,082 patients enrolled in the GSR-ET from May 2015 to December 2021, n = 2,013 patients who died during hospital stay after MT of LVO were excluded. In addition, patients with unknown survival status at discharge or 90 day follow-up (n = 3,125) and patients who were discharged from the MT-treating hospital to another hospital (n = 1,426) were excluded, because in-house mortality could not be differentiated from mortality after discharge in these patients. A comparison of patient, stroke and treatment characteristics of excluded patients with the study population is stated in table S1.

### Primary outcome and comparison groups

The primary outcome parameter was death at 90 day follow-up. Group comparison was performed for deceased patients (n = 757) versus patients alive at 90-day follow-up (n = 5,761), see also supplemental figure S1. Secondary outcomes were mortality rates in pre-defined subgroups depending on age, admission National Institute of Health Stroke scale (NIHSS), and NIHSS or modified Rankin scale (mRS) at discharge.

### Statistical analysis

Data is presented as median and interquartile range (IQR) or proportions (categorical variables), if not indicated otherwise. Group comparison on univariate level was performed by Mann–Whitney U test or chi-square test as appropriate. Multiple logistic regression analysis on the basis of complete data sets on the analysed outcome and predictor variables was conducted to identify adjusted odds ratio of 3-month mortality in patients with nonfatal LVO treated by MT. The following covariates were included in the model, based on a *p*-value < 0.1 in univariate group comparison: age, sex, pre-stroke living status, premorbid disability (mRS > 2), cardiovascular risk factors (diabetes mellitus, atrial fibrillation, arterial hypertension, current smoker), prior antiplatelets, prior anticoagulation, admission NIHSS, occluded vessel vertebrobasilar arteries, occluded vessel posterior cerebral artery, stroke aetiology, symptom onset/last seen well-to-admission, intravenous thrombolysis, general anaesthesia, successful recanalization (Thrombolysis in cerebral infarction scale [TICI] 2b-3), intracerebral haemorrhage, myocardial infarction during hospital stay, duration of hospital stay, discharge NIHSS, discharge mRS, discharge destination. Linearity between the continuous and quasi-continuous independent variables and the logit of the dependent variable was assessed using the Box-Tidwell procedure. Discharge NIHSS and discharge mRS were dichotomized according to optimal criterion value by Youden index calculation for the respective variable and the primary outcome (mRS > 3, NIHSS > 7). Following this procedure, all non-categorical variables were found to follow a linear relationship. Significance of predictive capacity of the multiple logistic regression model was assessed by Nagelkerke’s R^2^. Goodness of fit was assessed using the Hosmer–Lemeshow test, indicating a good model fit for *p* > 0.05. For multicollinearity diagnostics, we calculated variance inflation factors, assuming no relevant multicollinearity for values < 10. A significant difference was considered for *p* < 0.05 in all analyses. Statistical analyses were performed using SPSS® (Version 29, IBM®, Armonk, NY, USA).

## Results

### Univariate group comparison depending on survival status at 90 day follow-up

#### Baseline and stroke characteristics

**Fig. 1:**
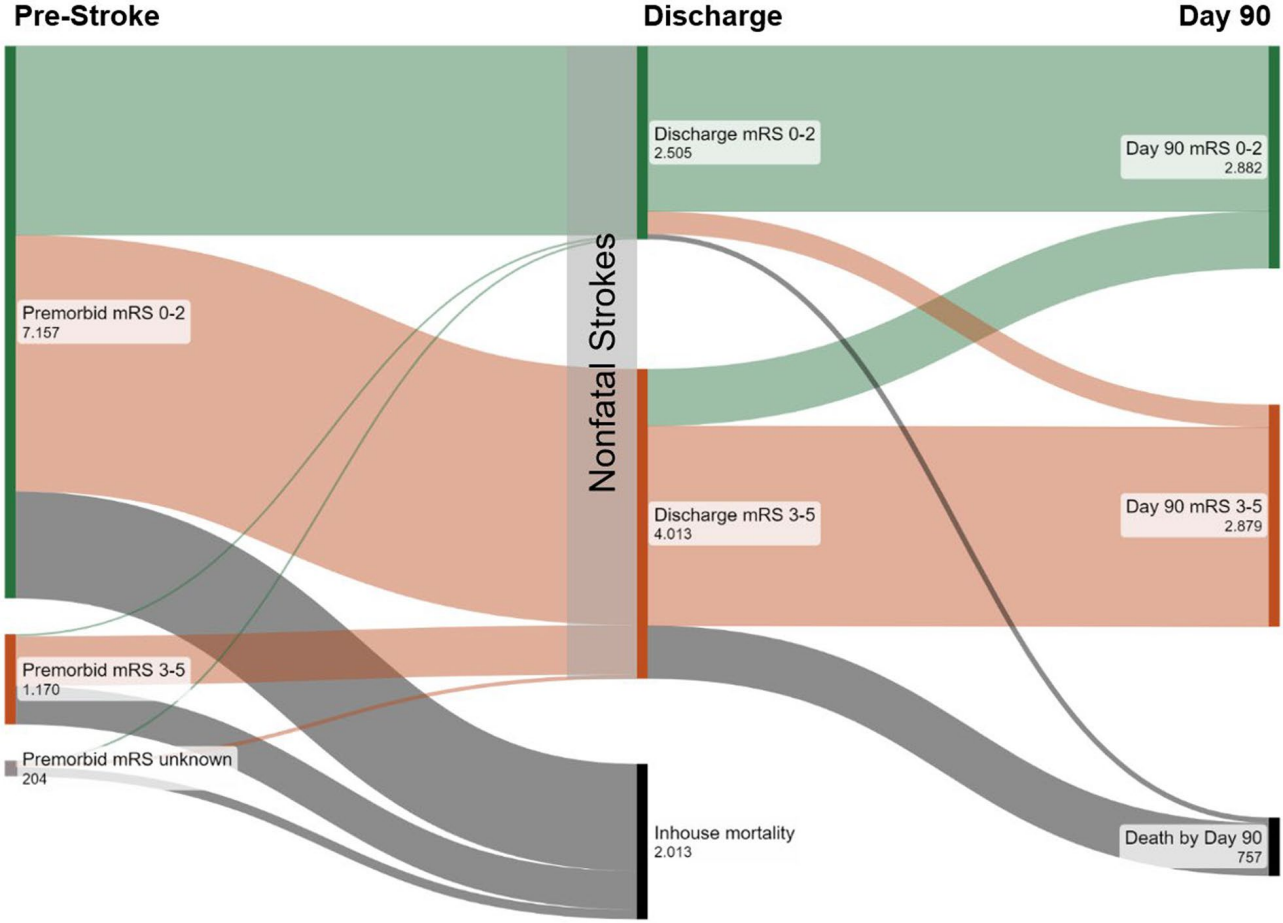
Premorbid functional status and outcome at discharge and 90 day follow-up after mechanical thrombectomy. Abbreviations: mRS: modified Rankin Scale

**Fig. 2:**
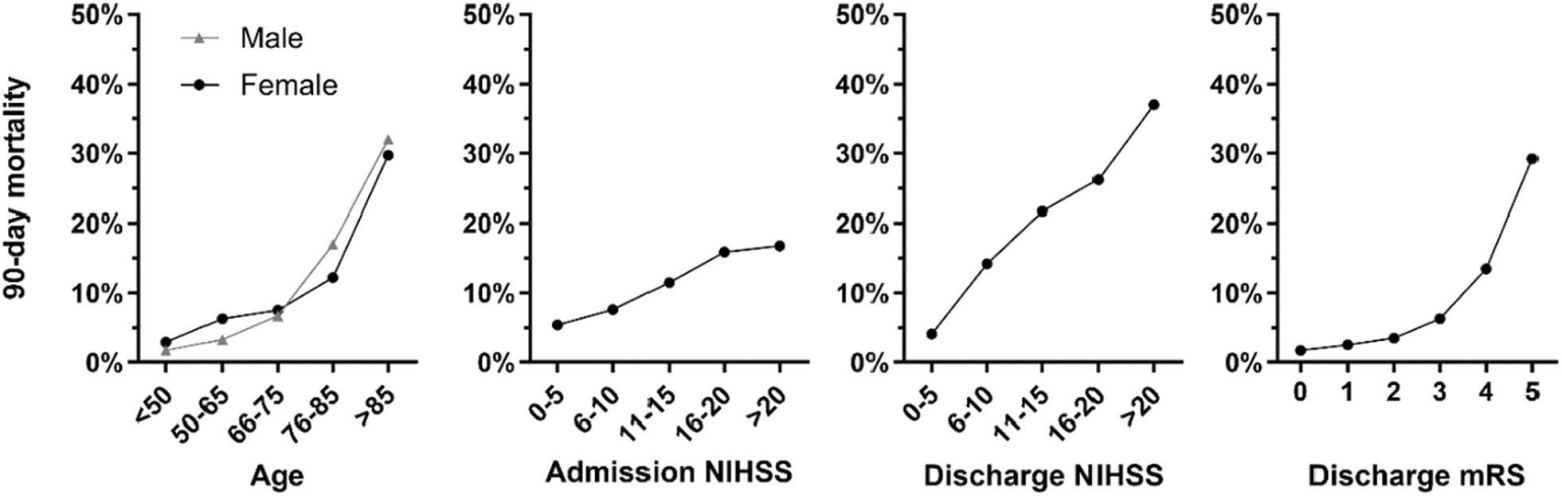
90-day mortality in nonfatal stroke treated by mechanical thrombectomy depending on age, stroke severity and functional outcome at discharge. Abbreviations: NIHSS: National Institutes of Health Stroke Scale, mRS: modified Rankin Scale

6,518 patients with nonfatal stroke treated by MT were included in our analysis (median age 75.0 years, 49.5% female). Of all patients with nonfatal stroke at discharge from treating hospital, 11.6% (n = 757) were deceased by 90-day follow-up (see Fig. 1). Correspondingly, patients with nonfatal stroke at discharge from treating hospital who had lethal outcome by day 90 comprise 27.3% of all patients with lethal outcome by day 90 (cumulative deaths in-hospital and after discharge, n = 2,770). For sensitivity analysis of 90 day mortality rates in nonfatal LVO by variation of survival status in patients with unknown survival status, excluded from the primary analysis, see table S2. In univariate group comparison (see Table 1), patients deceased between hospital discharge and day 90 were older (median age: 82 [IQR: 76–87] versus 74 [63–81], *p *< 0.001), more often female, had higher share of premorbid disability (26.4% versus 8.3%, *p* < 0.001) and more frequently had nursing care before LVO than patients who were alive at day 90. Cerebrovascular risk factors (arterial hypertension, diabetes mellitus and atrial fibrillation) were more frequent in patients with lethal outcome, whereas active smoking was more common in patients alive at 3-month follow up and dyslipidemia was equally present. Baseline medication with platelet inhibition and anticoagulation was more frequent in patients deceased by day 90. Deceased patients had higher stroke severity (admission NIHSS 16 [11.75–19] versus 13 [8–17], *p* < 0.001) and differing distribution of stroke etiology (more cardioembolic strokes), whereas the site of occluded vessels only differed for vertebrobasilar stroke (less frequent in patients with nonfatal stroke deceased by day 90). Mortality rates in subgroups of patients depending on age and admission NIHSS are illustrated in Fig. 2, showing a substantially increased mortality rate in patients aged > 85 years (males: 32.0%, females: 29.7%) and only moderate increase of mortality rates with increasing admission NIHSS.

**Table 1:** Baseline and stroke characteristics of patients with nonfatal stroke depending on survival status 3 months after mechanical thrombectomy

Variable	Deceased by day 90 (n = 757)	Alive at day 90 (n = 5,761)	*p*-value
Age	82 (76–87) (n = 756)	74 (63–81) (n = 5,757)	** < 0.001**
Female Sex	55.2% (418/757)	48.8% (2,811/5,759)	** < 0.001**
Premorbid disability (mRS 3–5)	26.4% (193/732)	8.3% (475/5,705)	** < 0.001**
Pre-stroke living status - Independent at home - Nursing at home - Nursing home	72.9% (543/733) 10.1% (74/733) 17.1% (125/733)	92.6% (5,266/5,675) 2.7% (153/5,675) 4.7% (266/5,675)	** < 0.001**
*Cardiovascular risk factors*			
Arterial hypertension	82.2% (620/754)	74.9% (4,299/5742)	** < 0.001**
Diabetes mellitus	26.7% (202/756)	20.4% (1,175/5,746)	** < 0.001**
Dyslipidaemia	45.1% (340/754)	43.3% (2,482/5,738)	0.339
Atrial fibrillation	55.5% (417/752)	38.3% (2,197/5,734)	** < 0.001**
Smoker (current)	11.7% (82/702)	17.9% (976/5,455)	** < 0.001**
*Baseline Medication*			
Anticoagulation	34.0% (253/744)	20.9% (1,189/5,696)	** < 0.001**
Platelet inhibition	33.7% (251/1,658)	29.1% (1,658/5,696)	**0.009**
*Stroke characteristics*			
NIHSS on admission	16 (11.75–19) (n = 746)	13 (8–17) (n = 5,722)	** < 0.001**
*Location of occlusion*			
Carotid artery	22.2% (165/743)	23.5% (1,335/5,670)	0.418
Anterior cerebral artery	3.4% (25/743)	2.4% (137/5,670)	0.121
Middle cerebral artery M1-segment	53.7% (399/743)	52.3% (2,964/5,670)	0.464
Middle cerebral artery M2-segment	24.1% (179/743)	24.1% (1,364/5,670)	0.983
Posterior cerebral artery	2.2% (16/743)	3.3% (187/5,670)	0.094
Vertebrobasilar arteries	6.5% (48/743)	9.0% (508/5,670)	**0.023**
Stroke aetiology - Large artery atherosclerosis - Cardioembolism - Dissection - Other - Undetermined	17.9% (135/756) 62.2% (470/756) 0.1% (1/1756) 4.0% (30/756) 15.9% (120/756)	27.8% (1,600/5,749) 47.9% (2,752/5,749) 2.4% (138/5,749) 4.5% (259/5,749) 17.4% (1,000/5,749)	** < 0.001**

Data are presented as percentage (absolute number) except for age and NIHSS on admission: median (IQR). Abbreviations: mRS: modified Rankin Scale; NIHSS: National Institutes of Health Stroke Scale

#### Treatment and outcome parameters

Patients who were alive at 90-day follow-up were more frequently treated by bridging intravenous thrombolysis (IVT) (52.6% versus 37.9%, *p* < 0.001) and had shorter duration from symptom onset to admission. Successful recanalization (TICI 2b-3) was more frequent in survivors (89.3% versus 82.0%, *p* < 0.001). Complications during hospital stay were balanced between both groups, except for intracerebral hemorrhage, which was more frequent in deceased patients. 3-month survivors had largely lower persisting neurological deficits at discharge from treating hospital (median NIHSS 4 [1–10] versus 12 [7–17], *p* < 0.001; median mRS 3 [1–4] versus 5 [4–5], *p* < 0.001), see Table 2 for more details. Mortality rates depending on NIHSS and mRS at discharge are illustrated in Fig. 2, showing a continuous increase of 3-month mortality with increasing discharge NIHSS and a steep increase of mortality rates in patients with discharge mRS of 4 (13.5%) and 5 (29.2%).

**Table 2:** Treatment characteristics and outcome parameters

Variable	Deceased by day 90 (n = 757)	Alive at day 90 (n = 5,761)	*p*-value
*Treatment characteristics*			
Intravenous thrombolysis	37.9% (286/754)	52.6% (3,015/5,736)	** < 0.001**
Primary admission at MT site	61.0% (440/721)	62.4% (3,432/5,503)	0.485
Symptom onset/Last seen well-to-admission (minutes)	196 (86–438) (n = 675)	168 (72–341) (n = 5,229)	** < 0.001**
Door-to-groin puncture (minutes)	71 (47–100) (n = 715)	69 (47–98) (n = 5,469)	0.379
General anaesthesia during MT	76.7% (562/733)	73.6% (4,082/5,549)	0.072
*Complications during hospital stay*			
Intracerebral haemorrhage	17.6% (132/750)	12.2% (693/5,701)	** < 0.001**
Device malfunction	0.7% (5/750)	0.3% (17/5,695)	0.104
Dissection/Perforation	2.1% (16/750)	2.6% (147/5,695)	0.463
Clot migration/embolization	3.2% (24/750)	3.7% (209/5,695)	0.517
Vasospasm	3.7% (28/750)	4.5% (255/5,695)	0.350
Malignant media infarction	1.3% (10/755)	2.0% (112/5,723)	0.229
Myocardial infarction	1.7% (13/755)	1.0% (56/5,723)	0.061
*Outcome parameters*			
Successful reperfusion (TICI 2b-3)	82.0% (609/743)	89.3% (5,022/5,625)	** < 0.001**
Duration of hospital stay (days)	11 (7–16) (n = 750)	10 (7–15) (n = 5,747)	** < 0.001**
NIHSS at discharge	12 (7–17) (n = 728)	4 (1–10) (n = 5,624)	** < 0.001**
mRS at discharge	5 (4–5) (n = 757)	3 (1–4) (n = 7,761)	** < 0.001**
Good outcome at discharge (mRS 0–2)	9.2% (70/757)	42.3% (2,435/5,761)	** < 0.001**
Excellent outcome at discharge (mRS 0–1)	4.5% (34/757)	25.0% (1,441/5,761)	** < 0.001**
Discharge destination - Home - Neurorehabilitation - Nursing home	13.2% (98/741) 70.9% (525/741) 15.9% (118/741)	31.0% (1,772/5,714) 66.1% (3,776/5,714) 2.9% (166/5,714)	** < 0.001**

Data are presented as percentage (absolute number) except for time metrics, NIHSS and mRS at discharge and duration of hospital stay: median (IQR). Abbreviations: mRS: modified Rankin Scale; MT: mechanical thrombectomy; NIHSS: National Institutes of Health Stroke Scale; TICI: Thrombolysis in cerebral infarction scale

### Independent predictors of 3 month mortality in nonfatal stroke treated by mechanical thrombectomy

**Fig. 3:**
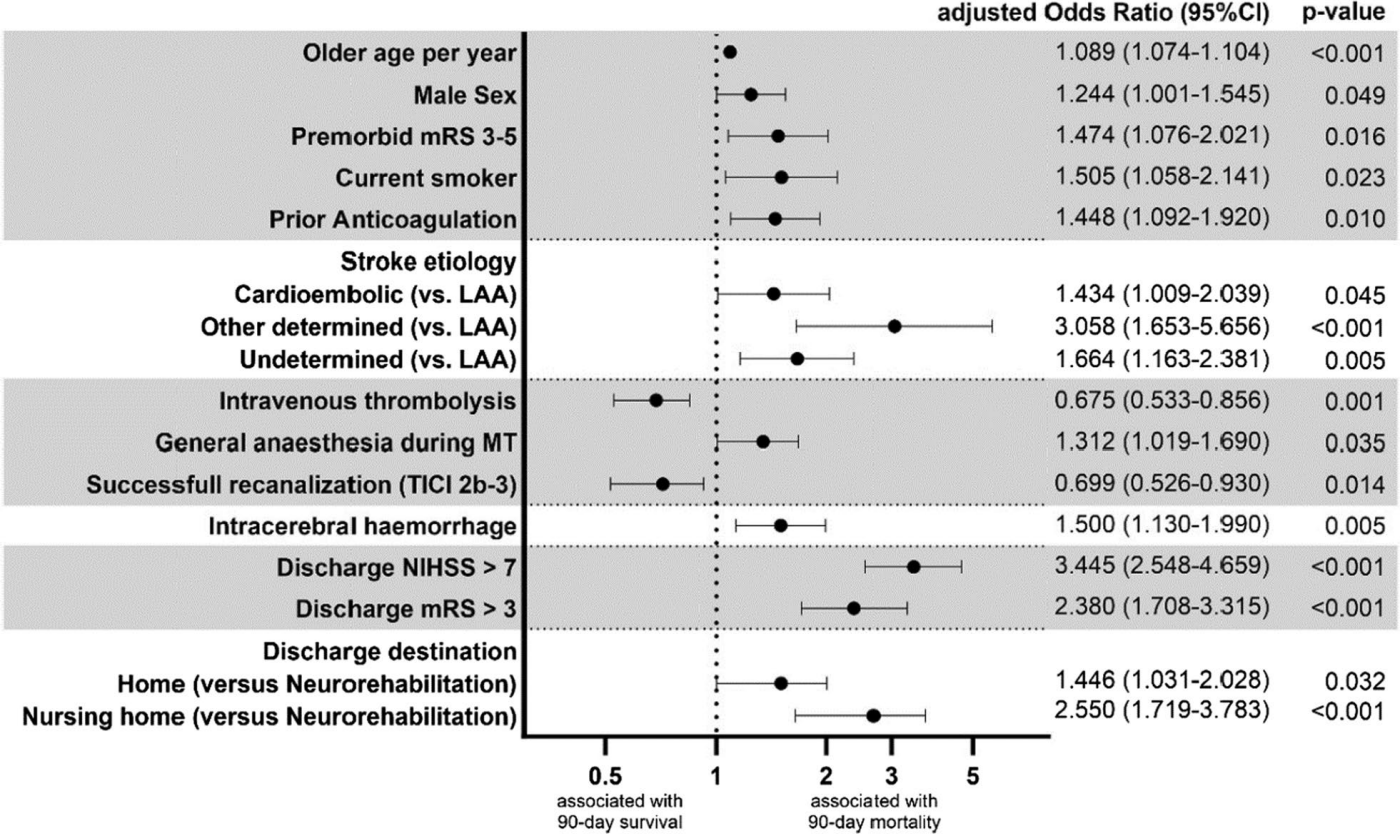
Independent predictors of 90-day mortality in nonfatal stroke treated by mechanical thrombectomy resulting from multiple logistic regression modelling (Nagelkerkes R^2^ = 0.336, *p* < 0.001). Abbreviations: mRS: modified Rankin Scale, LAA: large artery atherosclerosis, MT: mechanical thrombectomy, TICI: Thrombolysis in cerebral infarction scale, NIHSS: National Institutes of Health Stroke Scale

Baseline characteristics identified with multiple logistic regression analysis as independent predictors of 3-month mortality in patients discharged from hospital after MT (Fig. 3) were older age (adjusted odds ratio [aOR]: 1.089 [95% confidence interval: 1.074-1-104], *p* < 0.001), male sex (aOR: 1.244 [1.001-1-545], *p* = 0.049) and premorbid disability (aOR: 1.474 [1.076–2.021], *p* = 0.016). Of cerebrovascular risk factors, only active smoking was independently associated with lethal outcome (aOR: 1.505 [1.058-2.141], *p* = 0.023). Prior anticoagulation was identified as an independent predictor of 3-month mortality (aOR: 1.448 [1.092–1.920], *p* = 0.010). With regard to stroke etiology, large artery atherosclerosis was associated with survival as compared to all other etiologies, whereas ‘other determined’ etiology depicted the least favorable odds for survival. Both bridging IVT and successful recanalization were independent predictors of survival with comparable odds (IVT: aOR: 0.675 [0.533–0.868], p = 0.001; successful recanalization: aOR: 0.699 [0.526–0.930, p = 0.014). General anesthesia during MT was independently associated with lethal outcome (aOR: 1.312 [1.019–1-690], *p* = 0.035) as was any intracerebral hemorrhage (aOR: 1.500 [1.130–1.990], *p* = 0.005). Worse functional outcome at discharge was strongly associated with mortality (discharge NIHSS > 7: aOR: 3.445 [2.548–4.659], *p* < 0.001; discharge mRS > 3: aOR: 2.380 [1.708–3.315], *p* < 0.001). Of all discharge destinations (home, neurorehabilitation, nursing home), neurorehabilitation was associated with highest odds for 3 month survival.

## Discussion

Within our study, we show that more than a quarter of patients with lethal outcome three months after MT of LVO die only after discharge from the primarily treating hospital. With more than 10% of patients discharged alive from treating hospital being deceased by 3-month follow-up, mortality after nonfatal LVO is not rare. Therefore, predictors of mortality after nonfatal LVO are important to identify patients at high risk for lethal complications after discharge, for neurorehabilitative resource allocation and to support counselling about individual outcome prognosis and goals of care. We provide new insights into predictors of mortality in patients treated with MT, since predictors for mortality after stroke were so far mostly derived from mixed stroke cohorts or focussing on early mortality within the acute hospital stay of MT-treated patients [7, 19].

We report age to be a strong predictor of lethal outcome within three months after nonfatal stroke treated by MT. This is as expected and consistent with previous studies regarding in-hospital and cumulative 90 day mortality after MT [6, 7, 19, 28]. It is noteworthy that this relationship is largely skewed, with steeply increasing mortality rates in elderly patients > 85 years of age, of which roughly 30% have lethal outcome within three months after MT. Therefore, extracting patient preferences, goals of care and advance care planning should be included in counselling of these elderly patients, especially in case of further factors associated with increased risk for mortality being present. This need is supported by reports of low rates of advance directive completion among stroke survivors, and were shown to be positively influenced by discussion of advance care planning with a physician [15].

Premorbid disability (mRS > 2) was another factor assessed pre-interventional that was independently associated with 3-month mortality in nonfatal LVO in our analysis. It may support pre-interventional clinical decision-making, when taking into account patients’ values and goals of care. However, with a 1.5 fold increase of lethal outcome after hospital discharge, the strength of association with lethal outcome was much lower than for measures of post-intervention disability.

Of cerebrovascular risk factors, we report active smoking to be an independent predictor of mortality within three months after MT of nonfatal LVO. This finding urges smoking cessation efforts after MT of LVO, which is also supported by previous reports of reduced recurrent stroke rates and lower mortality throughout five years after acute cerebrovascular disease and smoking cessation [8].

Our study revealed prior anticoagulation as a predictive factor for lethal outcome within three months after nonfatal LVO. This finding needs further investigation and might be mediated by higher stroke recurrence rates in patients who have an LVO despite prior anticoagulation. In line with this, a large Swiss cohort study of direct oral anticoagulants in patients with ischemic stroke and atrial fibrillation recently reported a higher stroke recurrence rate in patients with prior anticoagulation [26].

Our analysis did not show independent association of LVO vessel territory with 90-day mortality after hospital discharge. This finding contrasts reports of predictors of in-house mortality after MT, in which especially carotid artery occlusion was described as a predictor of in-hospital mortality [17, 19]. Increased in-hospital mortality depending on vessel territory is likely explained by higher odds of malignant infarction and brain edema, a risk factor also described to be independently associated with in-house mortality after MT of LVO [3, 28]. In nonfatal LVO though, when discharged from hospital, our data do not support the relevance of vessel territory beyond persisting neurological deficits and further stated predictors in prognostication of 3 month mortality.

Both therapeutic interventions, application of bridging IVT and successful recanalization of LVO by MT, persisted as independent protective factors for 3-month survival in our analysis, which, importantly, adjusted also for time from symptom onset to hospital admission. MT under trial conditions often resulted in negative results for overall 90-day mortality. A systematic review of endovascular interventions for acute ischemic stroke including 19 RCTs points out a slightly decreased risk of death within the study period (relative risk: 0.85, 95% CI 0.75–0.97) in the treated population [22]. Our findings suggest that successful recanalization of LVO by MT not only reduces fatal stroke by prevention of malignant infarction, but is also associated with increased survival rates after discharge from treating hospital.

With regard to bridging IVT, trial data comparing MT alone with bridging IVT reported similar overall 90-day mortality in both groups. Our finding of bridging IVT being associated with 3-month survival in nonfatal LVO may result from a beneficial effect of bridging IVT for survival in patients who do not suffer acute complications, such as symptomatic intracranial haemorrhage, that occur more frequently with IVT [12] and are associated with in-hospital mortality [28]. Therefore, a risk-stratified approach regarding haemorrhagic complications in the decision for or against bridging IVT might maximize a potential beneficial effect of bridging IVT versus MT alone and warrants further investigation.

We report general anaesthesia during MT to be independently associated with lethal outcome after hospital discharge. This is of note, since RCT evidence regarding benefit and harm of anaesthesia choice during MT is still weak with inconsistent results [4, 24, 25]. A recently published multi-centre trial did not report differences in neither seven or 90-day mortality depending on anaesthesia type [5]. Further investigation of the effect of anaesthesia type on mortality in nonfatal stroke is warranted, especially in the context of potentially increased rates of primarily long-term prognosis-relevant complications, such as delirium, after general anaesthesia.

A large increase of odds for 3-month mortality in nonfatal LVO was observed for severe persisting functional deficits, with a discharge mRS of > 3 being associated with a 2.4-fold increase in death within three months after LVO and a discharge NIHSS of > 7 even increasing odds for lethal outcome 3.4-fold. As reported for age, we find it especially relevant for clinical practice that with mRS of 4 and 5 at discharge from treating hospital, a steep increase of mortality rate was observed (mRS 4: 13.5%, mRS 5: 29.2%). Therefore, intensified monitoring for treatable, possibly lethal complications, such as aspiration pneumonia, in patients with impaired mobility and self-care abilities is necessary.

In contrast, stroke severity (measured by admission NIHSS) did not reveal significant independent association with 3-month mortality, when adjusted for post-interventional deficits. This brings into question placing higher weight on stroke severity for pre-interventional decisions against (or for) MT in patients expected to have high chances for lethal outcome due to other factors (old age, premorbid disability).

Interestingly, discharge to a neurorehabilitative facility was independently associated with higher odds for 3-month survival in our analysis. This may, at least partly, be caused by gapless and intensified surveillance as well as timely treatment of medical complications in these patients in the early phase after hospital discharge. Thus, the benefit of neurorehabilitative practice should not be understated.

By analyzing an up-to-date large, prospective cohort of more than 13,000 MT procedures, our study benefits from a strong data foundation. However, there are several limitations to our findings. Due to the observational nature of our dataset, inference of causal relationships is not possible. We excluded patients, who were discharged to another hospital because in-hospital mortality was not distinguishable from lethal outcome after discharge in these patients. Therefore, our results are not transferable to patients who are referred from non-tertiary stroke centers only for MT and transferred back to the hospital of primary admission afterwards. Furthermore, our findings have limited transferability to patients that require transfer to another acute hospital for further reasons, such as non-neurological complications or comorbidities, which may have poorer prognosis. Our findings may also only be cautiously generalized to patients with premorbid disability, since we noted disproportionately high shares of missing outcome data in these patients, which has been reported before. [10] We analyzed a broad dataset on patient, stroke and treatment characteristics to identify independent predictors of 3-month mortality in patients who survive acute hospital stay after MT of LVO. However, we do not capture additional comorbidities that have been reported to be associated with mortality after ischemic stroke, such as malignant diseases and chronic cardiac disease. An association of such comorbidities with lethal outcome after discharge might be represented in our data by the observation of ‘other determined’ stroke etiology having the least favorable odds for 3-months survival. These will comprise also paraneoplastic coagulopathies and peri-interventional strokes during cardiovascular interventions. Additionally, we are missing detailed information on causes of death in deceased patients. We hypothesize that causes of death after hospital discharge are heterogeneous and also include a relevant proportion of non-stroke-related causes of death and deaths after therapy restrictions according to patient preferences such as withholding artificial nutrition, as has been reported for causes of death with longer distance to MT [28]. A more detailed evaluation of causes of death after hospital discharge could contribute to a better understanding of our findings and should be addressed in further studies.

Future studies should also address tools for multiparametric prognostic modelling in MT-treated patients surviving the acute hospital stay. This will increase prognostic accuracy and enable higher degrees of individualization of outcome prognosis at the time point of hospital discharge, relevant for complication management, neurorehabilitative resource allocation and assessment of palliative care needs. Besides clinical features, e.g. novel biomarkers of neuroaxonal damage are increasingly studied as prognostic features in acute ischemic stroke and could contribute to multimodal outcome prognostication. [23]. Multiparametric clinical scores are available already for pre-interventional outcome prognostication as well as for prediction of functional independence and mortality 24 h after MT [21], of which also in datasets of MT under routine-care conditions, the MR PREDICTS@24H was superior to outcome prognostication by early neurological outcome alone. At the same time, application of post-interventional scoring instruments in patients that survive the acute hospital stay resulted in diminished prognostic accuracy, which is expectable, since the scoring instruments were derived from mixed MT-treated cohorts. [11] Multiparametric outcome prognostication should therefore be tailored also to patients discharged from the acute hospital stay so that decisions about care pathways can be informed by accurate outcome prognostication.

## Conclusions

Lethal outcome after nonfatal LVO treated by MT is frequent, with more than 10% of patients discharged from treating hospital being deceased by 3-month follow-up. We show that patient, stroke and treatment characteristics enable individualization of lethal outcome prognostication. Our findings have direct implications for patients and physicians in clinical practice by improving identification of patients in need for intensified surveillance with regard to treatable medical complications after hospital discharge. Furthermore, since modifiable risk factors, such as active smoking, were associated with lethal outcome after nonfatal stroke, our data stress the importance of smoking cessation efforts to improve outcomes after MT of LVO. In other patient groups, which disproportionately presented with lethal outcome, such as patients older than 85 years and patients with discharge mRS of 5, the time point of hospital discharge after MT of LVO may be appropriate to address patient preferences, future goals of care and advance care planning in counselling of these patients and their relatives. Whenever possible, multiple prognostic factors should be considered in individual prognosis-making to account for variations within distinct patient groups, e.g., elderly patients. Addressing also palliative care needs seems even more important in the case of multiple factors associated with increased risk for mortality being present. Further studies are needed to elucidate the role of treatable and modifiable risk factors in lethal outcome after nonfatal LVO treated by MT in the future. Furthermore, future studies should develop multiparametric prognostic tools for MT-treated patients surviving the acute hospital stay in order to enable higher degrees of individualization of outcome prognosis in these patients.

## Supplementary Information

Additional file 1.

Additional file 2.

## Data Availability

The datasets used and/or analysed during the current study are available from the corresponding author on reasonable request.

## Abbreviations

aOR: Adjusted odds ratio
GSR-ET: German Stroke Registry—endovascular treatment
IVT: Intravenous thrombolysis
LVO: Large vessel occlusion
mRS: Modified Rankin scale
MT: Mechanical thrombectomy
NIHSS: National instuitute of health stroke scale
RCT: Randomized controlled trial
TICI: Thrombolysis in cerebral infarction scale
