# Supplementary material for: Mortality within three months after nonfatal ischemic stroke treated by mechanical thrombectomy in routine care—data from the German Stroke Registry
Source: Neurol Res Pract. 2025 Oct 1;7(1):71. doi: 10.1186/s42466-025-00427-7 (PMC12490031; doi:10.1186/s42466-025-00427-7)
Supplement: Supplementary file 1 — Additional file 1. [file 42466_2025_427_MOESM1_ESM.pptx]

## Slide 1
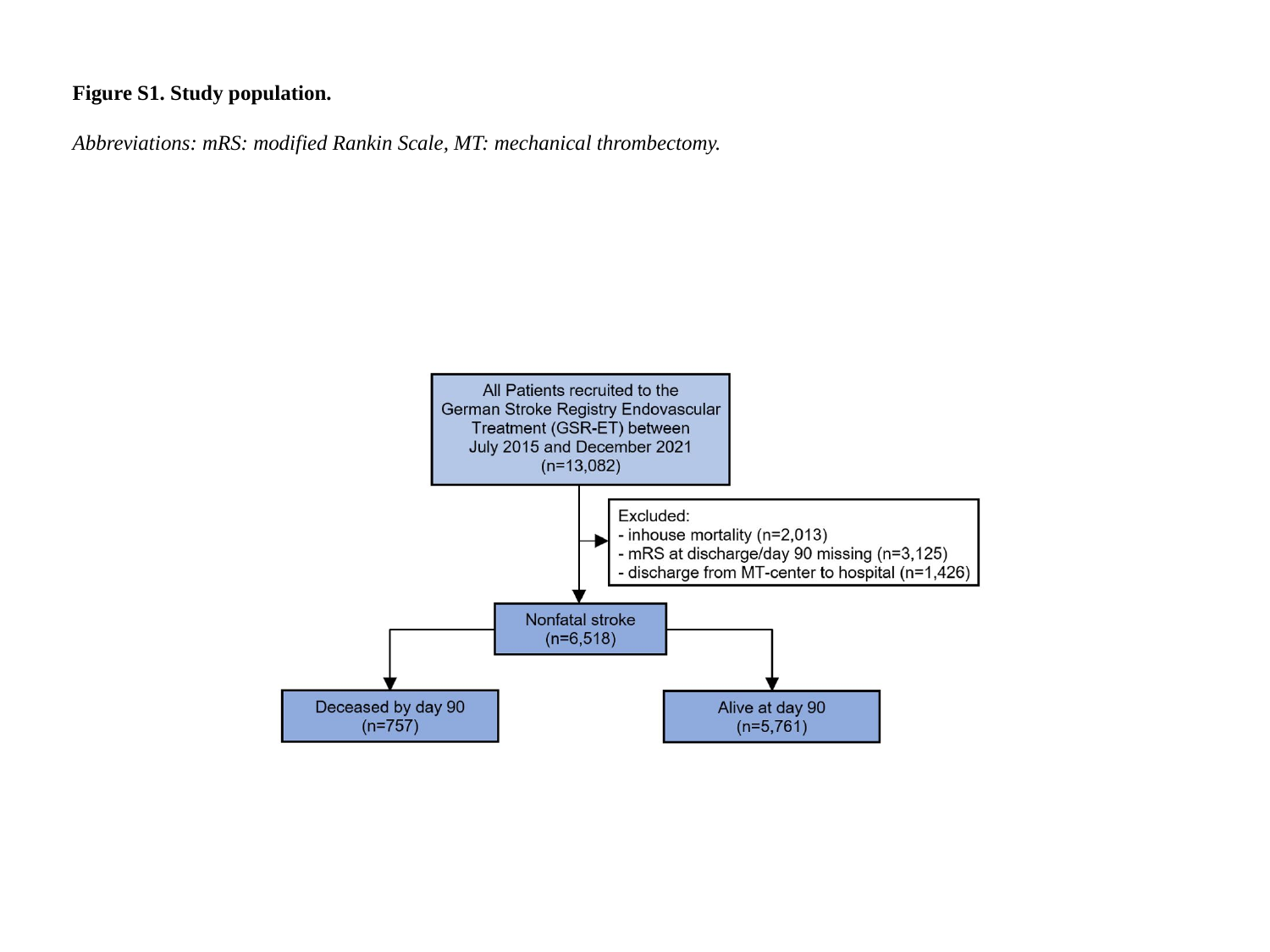

Figure S1. Study population.
Abbreviations: mRS: modified Rankin Scale, MT: mechanical thrombectomy.
