# Supplementary material for: Mortality within three months after nonfatal ischemic stroke treated by mechanical thrombectomy in routine care—data from the German Stroke Registry
Source: Neurol Res Pract. 2025 Oct 1;7(1):71. doi: 10.1186/s42466-025-00427-7 (PMC12490031; doi:10.1186/s42466-025-00427-7)
Supplement: Supplementary file 2 — Additional file 2. [file 42466_2025_427_MOESM2_ESM.docx]

**Table S1. Sensitivity analysis – Comparison of baseline, stroke and treatment characteristics of patients with nonfatal stroke (study cohort) and patients that were excluded due to absent mRS at discharge/ 90-day follow-up or discharge to another hospital.**

| Variable | Excluded patients  (n=4,317) | Nonfatal strokes (study cohort) (n=6,518) | p-value |
| --- | --- | --- | --- |
| Age | 76 (64-76) (n=4,311) | 75 (64-82) (n=6,513) | **0.013** |
| Female Sex | 51.0% (2,199/4,310) | 49,6% (3,229/6,516) | 0.135 |
| Premorbid disability (mRS 3-5) | 22.2% (797/3,598) | 10,4% (668/6,437) | **<0.001** |
| Pre-stroke living status   - Independent at home - Nursing at home - Nursing home | 90.7% (3,351/3,696) 4.3% (159/3,696) 5.0% (186/3,696) | 90.4% (5,790/6,408) 3,5% (227/6,408) 6,1% (391/6,408) | **0.016** |
| *Cardiovascular risk factors* |  |  |  |
| Arterial hypertension | 76.5% (2,777/3632) | 75,7% (4,919/6,496) | 0.406 |
| Diabetes mellitus | 22.3% (790/3,539) | 21.2% (1,377/6,502) | 0.183 |
| Dyslipidaemia | 39.7% (1,388/3,496) | 43.5% (2,822/6,492) | **<0.001** |
| Atrial fibrillation | 41.2% (1,453/3,524) | 40.3% (2,614/6,486) | 0.366 |
| Smoker (current) | 18.5% (592/3,202) | 17.2% (1058/6,157) | 0.116 |
| *Baseline Medication* |  |  |  |
| Anticoagulation | 26.5% (903/3,409) | 22.4% (1,442/6,440) | **<0.001** |
| Platelet inhibition | 29.7% (1,014/3,409) | 29.6% (1,909/6,440) | 0.916 |
| *Stroke characteristics* |  |  |  |
| NIHSS on admission | 14 (8-18) (n=3,700) | 13 (8-18) (n=6,468) | 0.175 |
| *Location of occlusion* |  |  |  |
| Carotid artery | 24.6% (1.009/4.101) | 23.4% (1,500/6,413) | 0.160 |
| Anterior cerebral artery | 1.9% (78/4,104) | 2.5% (162/6,413) | **0.036** |
| Middle cerebral artery  M1-segment | 54.6% (2,242/4,101) | 52.4% (3,363/6,413) | **0.028** |
| Middle cerebral artery  M2-segment | 22.7% (930/4,104) | 24.1% (1,543/6,413) | 0.099 |
| Posterior cerebral artery | 2.5% (103/4,104) | 3.2% (203/6,413) | 0.051 |
| Vertebrobasilar arteries | 9.7% (399/4,101) | 8.7% (556/6,413) | 0.067 |
| Stroke aetiology   - Large artery atherosclerosis - Cardioembolism - Dissection - Other - Undetermined | 27.4% (1000/3,656) 50.1% (1,830/3,656) 1.4% (51/3,656) 5.4% (196/3,656) 15.8% (579/3,656) | 26.7% (1,735/6,505) 49.5% (3,222/6,505) 2.1% (139/6,505) 4.4% (289/6,505) 17.2% (1,120/6,505) | **0.007** |
| *Treatment characteristics* |  |  |  |
| Intravenous thrombolysis | 45.9% (1,930/4,202) | 50.9% (3,301/6,490) | **<0.001** |
| Primary admission at MT site | 45.5% (2,288/4,048) | 62.2% (3,872/6,224) | **<0.001** |
| Symptom onset/Last seen well-to-admission (minutes) | 190 (82-352) (n=3,043) | 171 (73-350) (n=5,904) | **0.001** |
| Door-to-groin puncture (minutes) | 72 (45-106) (n=3,538) | 69 (47-99) (n=6,184) | **0.025** |
| Number of passages | 1 (1-3) (n=3,714) | 2 (1-3) (n=6,044) | 0.797 |
| General anaesthesia during MT | 82.5% (3,284/3,983) | 73.9% (4,644/6,282) | **<0.001** |
| *Complications during hospital stay* |  |  |  |
| Intracerebral haemorrhage | 8.7% (360/4,140) | 12.8% (825/6,451) | **<0.001** |
| Device malfunction | 0.3% (11/4,135) | 0.3% (22/6,445) | 0.498 |
| Dissection/Perforation | 2.5% (102/4,135) | 2.5% (163/6,445) | 0.841 |
| Clot migration/embolization | 3.1% (127/4,135) | 3.6% (233/6,445) | 0.132 |
| Vasospasm | 4.1% (171/4,135) | 4.4% (283/6,445) | 0.527 |
| Malignant media infarction | 2.0% (74/3,772) | 1.9% (122/6,478) | 0.780 |
| Myocardial infarction | 1.1% (40/3,772) | 1.1% (69/6,478) | 0.982 |
| *Outcome parameters* |  |  |  |
| Successful reperfusion (TICI 2b-3) | 88.3% (3,589/4,063) | 88.4% (5,631/6,368) | 0.885 |
| Duration of hospital stay (days) | 7 (4-12) (n=3,665) | 10 (7-15) (n=6,497) | **<0.001** |

*Table legend: Data are presented as percentage (absolute number) except for age and NIHSS on admission: median (IQR). Abbreviations: NIHSS: National Institutes of Health Stroke Scale.*

**Table S2. Sensitivity analysis of 90-day mortality rate in patients with nonfatal LVO treated by mechanical thrombectomy: minimum-maximum bias assumptions by alteration of survival status in patients excluded from the primary analysis.**

| **Scenario** | **mRS discharge unknown** | **mRS day 90 unknown** | **Transfer to another hospital** | **90-day mortality rate of nonfatal LVO**  **(deceased day 90/ discharge survivors)** |
| --- | --- | --- | --- | --- |
| Original manuscript | excluded | excluded | excluded | **11.6%** (757/6,518) |
| 1 | In-house mortality | Day 90 deceased | In-house mortality, if day 90 mRS=6 | **22.4%** (2,070/9,231) |
| 2 | In-house mortality | Day 90 deceased | Mortality after discharge, if day 90 mRS=6 | **26.9%** (2,641/9,802) |
| 3 | In-house mortality | Day 90 alive | In-house mortality, if day 90 mRS=6 | **8.2%** (757/9,231) |
| 4 | In-house mortality | Day 90 alive | Mortality after discharge, if day 90 mRS=6 | **13.5%** (1,328/9,802) |
| 5 | Discharged alive | Day 90 deceased | In-house mortality, if day 90 mRS=6 | **31.8%** (3,337/10,498) |
| 6 | Discharged alive | Day 90 deceased | Mortality after discharge, if day 90 mRS=6 | **35.3%** (3,908/11,069) |
| 7 | Discharged alive | Day 90 alive | In-house mortality, if day 90 mRS=6 | **7.2%** (757/10,498) |
| 8 | Discharged alive | Day 90 alive | Mortality after discharge, if day 90 mRS=6 | **12.0%** (1,328/11,069) |
